# The Effect of Training Inhalation Technique with or without Spacer on Maximum Expiratory Flow Rate and Inhaler Usage Skills in Asthmatic Patients: A Randomized Controlled Trial

**Published:** 2014-10

**Authors:** Hashem Rahmati, Fatemeh Ansarfard, Fariba Ghodsbin, Mohammad Ali Ghayumi, Mehrab Sayadi

**Affiliations:** 1 Department of Medical Surgical Nursing, School of Nursing and Midwifery, Shiraz University of Medical Sciences, Shiraz, Iran;; 2 Community based Psychiatric Care Research Center, Department of Community Health Nursing, School of Nursing and Midwifery, Shiraz University of Medical Sciences, Shiraz, Iran;; 3 Department of Internal Medicine, School of Medicine, Shiraz University of Medical Sciences, Shiraz, Iran;; 4 Cardiovascular Research Center, Shiraz University of Medical Sciences, Shiraz, Iran

**Keywords:** Asthma, Metered Dose Inhaler, Peak Expiratory Flow Rate, Spacer

## Abstract

**Background:** The most common treatment for asthma is transferring the drug into the lungs by inhaler devices. Besides, correct use of inhaled medication is required for effectiveness of pharmacotherapy. Thus, it is necessary to train the patients how to use Metered Dose Inhaler (MDI). This study aimed to determine the effect of training about MDI usage with or without spacer on maximum expiratory flow rate and inhaler usage skills in asthmatic patients.

**Methods: **This randomized clinical trial was conducted on 90 asthmatic patients who were randomly divided into inhalation technique group with spacer, inhalation technique group without spacer, and a control group. Then, the Peak Expiratory Flow Rate (PEFR) was measured using a peak flow meter, as a basic test. In addition, the patients’ functional skills of inhalation technique were assessed using two checklists. Afterwards, 3 sessions of training were arranged for both groups. PEFR and the ability to use the MDI were evaluated immediately and 1 month after the intervention. Finally, the data were entered into the SPSS statistical software (v. 18) and analyzed using independent t-test and repeated measures ANOVA.

**Results:** After the intervention, MDI usage skills improved in the two intervention groups compared to the control group (P<0.001). In addition, a significant difference was found between the intervention groups and the control group regarding the mean of PEFR after the intervention (P<0.001). However, no significant difference was observed between the two intervention groups (P=0.556).

**Conclusion: **According to the results, providing appropriate training for asthmatic patients increased MDI usage skills, and both methods of inhalation (with or without spacer) could improve the PEFR among the patients.

**Trial Registration Number:** IRCT2013091514666N1

## Introduction

Asthma is a chronic inflammatory disease that causes airways hypersensitivity, mucosal edema, and mucus production. This inflammation leads to recurrent episodes of asthma symptoms; i.e., cough, chest tightness, wheezing, and dyspnea.^1^ According to the research findings, 5% of the population around the world suffers from asthma.^2^ In other words, about 300 million people around the world are suffering from this disease and this figure is expected to increase to 400 million people by 2020.^3^ Besides, 250000 deaths annually occur due to asthma all over the world.^4^ In Iran, nearly 6.5 million people suffer from this disease.^5^ Metered Dose Inhalers (MDIs) are the most common drug-delivery systems for aerosolized therapy in asthmatic patients.^6^ “The major advantage of inhalation therapy is that the drugs are delivered directly into the airways, achieving higher local concentrations with significantly less risk of systemic side effects”.^7^ Using MDI requires some steps to be taken which have to be covered properly, because performance of one or more of these steps in a wrong way can affect the administration and effect of the drug on the body.^7^ These mistakes can also lead to a decrease in the therapeutic effects, weak control of the symptoms, and poor management of the disease.^8^^,^^9^ The rate of drug distribution in the lungs has been reported to be 10% which is because of the improper usage of MDI by asthmatic patients.^10^ Some researchers have shown improper usage of the MDI by asthmatic patients as one of the most significant problems of their treatment. According to these studies, the percentage of the individuals who use MDI appropriately in Iran was considerably lower compared to the modern countries.^11^ Several studies have demonstrated that using MDI connected to a spacer could cause a better drug distribution in the lungs with less oropharyngeal deposition of medication.^12^ Yet, training patients regarding the proper use of any facility is necessary to increase the therapeutic benefits.^13^

Peak flow meter is a device used to measure the Peak Expiratory Flow Rate (PEFR). PEFR has been accepted as an independent measure of lung function.^1^^,^^14^ In asthmatic patients, airway obstruction is measured by PEFR.^15^ Some studies have revealed that correct instruction of using MDI could improve bronchodilation.^16^ Patients might receive treatment, but without proper instruction and training regarding the correct inhalation techniques, the therapeutic benefit would be less than optimal.^17^ Training the asthmatic patients is a fundamental part of their management and this instruction is needed to achieve the necessary self-esteem, skills, and motivation for controlling the disease‌. Overall, using the MDI properly has a crucial role in effective treatment of asthma^18^^,^^19^ and, consequently, training the patients is essential to improve their treatment.^19^ “Furthermore, it has been established that inhaler technique training must be repeated regularly in order to maintain the correct technique”.^13^^,^^20^ “To acquire the skills for using these devices, patients must be adequately trained, and healthcare personnel are responsible for training the correct use of inhalation devices”.^21^

According to national and international findings regarding asthma, it is necessary to check and correct the inhalation techniques used by asthmatic patients.^7^Consequently, studies are recommended to be performed on assessment of the impact of patients’ instruction. Moreover, due to the increasing number of the individuals using MDI in a wrong way, it is important to evaluate the patients’ inhalation techniques because the best and only way of asthma treatment is using MDIs. In this way, the costs and mortality rate due to improper usage of the MDIs will decrease, as well. Thus, the present research aims to determine the effect of training about MDI usage with or without spacer on PEFR and inhaler usage skills in asthmatic patients.

## Materials and Methods

This Randomized clinical trial was carried out from April 2013 to may 2013 on asthmatic patients who had referred to the clinics affiliated to Shiraz University of Medical Science, Shiraz, Iran. According to the study objectives and the previous studies conducted on the issue and considering standard deviation of 7.2 and power of 90%, a 90-subject sample size (30 in each group) was determined for the study using the following formula:

$$n=\frac{2{\left({\text{Z}}_{1-\frac{\alpha }{2}}+{\text{Z}}_{1-\beta }\right)}^{2}{\text{S}}^{2}}{{\text{d}}^{2}}$$

**Figure 1:**
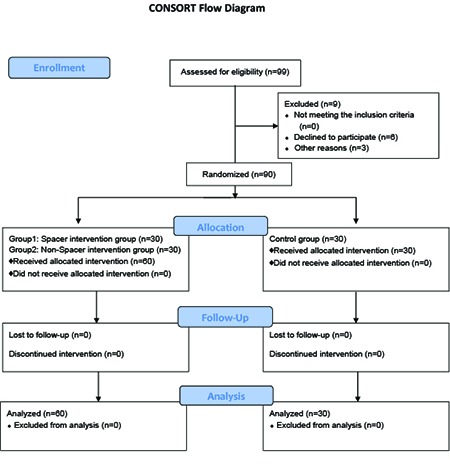
Diagram of the participants in the study

Overall, 99 subjects were assessed for eligibility. However, 9 subject were excluded from the study due to decline to participation in the study (n=6) and other reason (n=3). Therefore, the study was done on 90 subjects (30 in each group). It should be mentioned that none of the participants was excluded from the study during the follow up and data analysis. Figure 1 shows the diagram of the participants in this study. The inclusion criteria of the study were being 18 to 60 years old, having a past history of using Salbutamol MDI (manufactured by Sinadarou Company) for at least 3 months, and not participating in similar interventional programs. On the other hand, the exclusion criteria of the study were smoking, having an asthmatic attack, and not being willing to continue cooperation in the study.

After the study protocol was approved by the research Ethics Committee of the University, the researcher referred to Motahari and Faghihi clinics and after getting permission from the hospital authorities, 99 participants were selected through convenience sampling. After providing the participants with the necessary explanation about the research objectives, written informed consents were obtained from all the participants. Then, the subjects were randomly divided into two intervention groups and a control group using block randomization with a random sequence of 6 block sizes. It should be noted that the patients were aware of the reasons of the interventions and the research was not thus blinded. In the next step, the researcher observed and checked inhaler usage skills of the three groups to fill out the 11-item checklists. The two check lists were different. Therefore, the researcher asked the spacer group to show the inhalation technique with spacer. The non- spacer group, on the other hand, was required to show inhalation technique without spacer and the control group was required to show inhalation technique in both approaches. Afterwards, the two intervention groups were separately compared to the control group. The PEFR was measured for the three groups before and 15 min after inhaling 200µg salbutamol as the baseline test. The study data were collected using a demographic questionnaire made by the researcher, two 11-item checklists for checking MDI usage skills scored by 0 and 1 (attachment 1), and peak flow meter (Peak Pocket, made in England) for checking PEFR. In addition to reviewing the literature and references, the content validity of the check lists was reviewed and corrected by 4 professors of Shiraz School of Nursing and Midwifery. Besides, in order to determine the reliability of the checklists, the researcher observed and checked inhalation technique of 10 asthmatic patients and filled out the check lists. Then, another expert in this field observed and checked the inhalation technique in the same 10 patients and the correlation between the two observers was measured as 0.95 (P=0.63).

Three educational sessions, both theoretical and practical, were held for the two intervention groups. Inhalation techniques with and without spacer were instructed in the spacer group and non-spacer group, respectively. The educational classes were held by presenting a lecture, showing a power point, question and answer, and evaluating the participants at the end of the sessions. The content of the instructional sessions in the non-spacer group included the principles of asthma, importance and advantages of correct inhalation technique, training about inhalation technique without using spacer, and repetition and reinforcement of training that were presented in three session. Steps of inhalation technique without spacer consist of: 1) Take the MDI cover away and hold it straightly, 2) Shake the MDI, 3) Head backward and exhale, 4) Seal the mouthpiece against closed lips or 2-2.5 cm far from the mouth, 5) Push the button during inhalation, 6) Perform slow inhalation, 7) Coordinate between inhalation and actuation, 8) Continue slow and deep inhalation for 5–10 seconds, 9) Stop breathing for 5–10 seconds, 10) Exhale through pursed lips, 11) Wait for 1 minute before repeating the maneuver

Additionally, the content of the instructional sessions in the spacer group included asthma illness, importance of correct inhalation technique, using spacer connected to MDI, training about the correct inhalation technique with spacer, and repetition and reinforcement of training that were presented in three session. Steps of Inhalation technique with spacer consist of:

1) Take the MDI cover away and hold it straightly, 2) Shake the MDI, 3) Connect the MDI to spacer, 4) Head backward and exhale, 5) Insert the mouth part of the assistant in the mouth correctly, 6) Push the button during inhalation, 7) Perform slow inhalation, 8) Continue slow and deep inhalation for 5–10 seconds, 9) Stop breathing for 5–10 seconds, 10) Repeat slow and deep breathing in the assistant, 11) Wait for 1 minute before repeating the maneuver.

Therefore inhalation techniques with and without spacer were trained in the spacer and non-spacer groups, respectively, and the relevant instructional booklets were prepared. It should be mentioned that the time of the classes was adjusted according to the intervention groups participants’ comfort. The control group, however, did not receive any interventions. The two intervention groups’ subjects were emphasized not to explain the educational program and give any information to the other subjects. Then, using the similar method to that before the intervention, the patients’ ability to use inhalation MDIs correctly and their PEFR were evaluated immediately and one month after the end of the training. Finally, the data were entered into the SPSS statistical software (v. 18) and analyzed using independent T-test, chi-square, Fisher exact test, and ANOVA. 

## Results

The results of Chi-square test and Fisher’s exact test showed no significant differences among the three groups regarding the demographic variables, including age, gender, education level, employment status, duration of using MDI, and duration of suffering from asthma (table 1).

**Table 1:** Demographic characteristics of the patients in the three groups

**Demographic variables**		**Control group**	**Non-spacer group**	**Spacer group**	**P value**
		**N (%)**	**N (%)**	**N (%)**	
Gender	Male	13 (43.3)	8 (26.7)	10 (33.3)	0.430
	Female	17 (56.7)	22 (73.3)	20 (66.7)	
Education level	Elementary school	10 (33.3)	2 (6.7)	5 (16.7)	0.102
	Middle school	4 (13.3)	7 (23.3)	2 (6.7)	
	High school and diploma	10 (33.3)	11 (36.6)	15 (50)	
	Academic	6 (20)	10 (33.3)	8 (26.7)	
Employment status	Homemaker	14 (46.7)	17 (56.7)	10 (33.3)	0.062
	Worker	1 (3.3)	1 (3.3)	0 (0)	
	Student	1 (3.3)	0 (0)	6 (20)	
	Self-employed	6 (20)	1 (3.3)	5 (16.7)	
	Employee and retiree	8 (26.7)	11 (36.7)	9 (30)	
Mean age (yrs±SD)		44.7±10.8	41.5±9.1	42.7±13.8	0.550
Duration of suffering from asthma (yrs±SD)		2.8±0.94	2.7±0.95	2.6±0.85	0.690
Duration of using MDI (yrs±SD)		2.7±0.91	2.6±0.95	2.4±0.97	0.591

The results of this study concerning the increase in PEFR in response to inhalation of 200 microgram Salbutamol MDI have been presented in table 2. The results of ANOVA revealed no significant difference among the three groups regarding PEFR before the intervention (P=0.38). However, a significant difference was observed between the control and the intervention groups in this regard immediately and a month after the intervention (P=0.000).

**Table 2:** Comparison of the mean changes in the subjects’ PEFR in the two intervention groups and the control group

**(PEFR changes in response to inhalation of 200µg salbutamol)** **Mean±SD**			
** Time** **Groups**	**Before the intervention**	**Immediately ** **‌** **after the intervention**	**One month after the intervention**
Control	19.2±16.4	18.8±10.4	20±9.4
Non-spacer	23.6±10.8	79±20.3	73.1±18.6
Spacer	23±11.3	86.1±27.1	81.6±25.5
P value	0.380	<0.05*	<0.05*

*P values<0.05 were considered as statistically significant

Based on the results of Dunnet T3 test presented in table 3, a significant improvement was observed in PEFR in both intervention groups compared to the control group immediately ‌after the intervention (P=0.000). Although the rate of changes was higher in the spacer group compared to the non-spacer group, the difference was not statistically significant (P=0.5). A month after the intervention, there was still an improvement in the group using spacer (P=0.000); however, no significant difference was seen again (P=0.26).

**Table 3:** Comparison of the mean difference of increase in the subjects’ PEFR in the   intervention and control groups

**Time**	**Immediately** **‌** ** after the intervention**			**One month after the intervention**	
**Group**	**Comparison with the other two** **‌** **groups**	**Mean difference**	**P value**	**Mean difference**	**P value**
Spacer	Control	67.3	<0.05*	61.6	<0.05*
	Non-space	7.1	0.556	8.5	0.26
Non- spacer	Spacer	-7.1	0.556	-8.5	0.26
	Control	60.1	<0.05*	53.1	<0.05*
Control	Spacer	-67.3	<0.05*	-61.6	<0.05*
	Non-spacer	-60.1	<0.05*	-53.1	<0.05*

*P values<0.05 were considered as statistically significant

Assessment of the inhalation technique before the intervention in the intervention and control groups indicated that the most important pitfall was inhaling the MDI slowly. This problem was detected in 66% of the participants. Another problem was about how to continue slow and deep inhalation for 5–10 seconds followed by how to coordinate between inhalation and actuation (74% and 66.7%, respectively).

On the other hand, the least important problem was about the second step; i.e., how to shake the MDI, and holding the MDI 2–2.5 cm far from the mouth (31.7% and 25%, respectively). 

The results of comparison of the inhalation technique in the control and non-spacer groups have been presented in table 4. The results of independent T-test showed no significant difference between the non-spacer and the control group concerning MDI usage skills before the intervention (P=0.54). However, a significant difference was found between the two groups in this regard immediately and one month after the intervention (P=0.000) (table 4).

**Table 4:** Comparison of the subjects’ means of inhalation technique skills in non-spacer and control groups

**Time**	**Before the intervention**	**Immediately** **‌** ** after the intervention**	**1 month after the intervention**
**Group **	**Mean±SD**	**Mean±SD**	**Mean±SD**
Non- spacer	6.2±1.37	10.7±0.65	9.93±1.01
Control	5.9±1.56	5.7±1.3	5.8±1.37
P value	0.540	<0.05*	<0.05*

*P values<0.05 were considered as statistically significant

The results of comparison of the inhalation technique in the control and spacer groups have been shown in table 5. The results demonstrated no significant difference between the spacer and the control group regarding MDI usage skills before the intervention (P=0.8). Nonetheless, a significant difference was observed between the two groups in this regard immediately and one month after the intervention (P=0.000).

**Table 5:** Comparison of the subjects’ means of inhalation technique skills in  spacer and control groups

**Time**	**Before the intervention**	**Immediately** **‌** ** after the intervention**	**1 month after the intervention**
**Group **	**Mean±SD**	**Mean±SD**	**Mean±SD**
Spacer	5.46±1.07	10.9±0.18	10.4±0.73
Control	5.53±1.27	5.43±1.1	5.16±1.1
P value	0.800	<0.05*	<0.05*

*P values<0.05 were considered as statistically significant.

## Discussion

The present research aimed to compare using MDI with or without spacer to determine the patients’ PEFR and ability to use the MDI. The study results showed that the most common problem was slow inhalation and coordination between inhalation and actuation. This finding is consistent with that of the study conducted by Bagherinesami.^5^ Similarly, the results of the study by AL Amoudi indicated that one of the biggest problems with MDI application was related to the slow and deep inhalation stage.^22^ It is obvious that problems in each step of MDI usage prevent proper drug transfer to the lungs and this is the reason why asthma is not controlled.

The current study results revealed an increase in the average rate of inhalation technique after the intervention, indicating the effectiveness of the training. In the same line, Hamelin’s study showed that proper instruction could improve inhalation techniques in the patients suffering from Chronic Obstructive Pulmonary Disease (COPD) and asthma.^7^ Another study also demonstrated that instruction of inhalation technique both with and without spacer could improve inhalation skills, which is in agreement with the findings of our study.^17^ However, no studies have been conducted on this issue in Iran, except for the one performed by Bagherinesami which indicated that written and oral instruction could help nurses use MDI correctly.^5^ In general, the most common method of treatment of inhalation diseases is using MDI which is not instructed completely to the patients. Also, the importance of patients’ instruction on the correct usage of MDI has often been underestimated.^22^ Thus, the patients who do not use MDIs correctly must be trained regularly until they learn the correct way of applying MDIs. Educational aids can also be effective in this process.^17^

The present study showed a significant improvement in the two intervention groups’ PEFR compared to the control group. However, no significant difference was observed between the two intervention groups in this regard. Therefore, both methods were useful for asthmatic patients and they could use either way to treat asthma. Similarly, few studies have indicated that instruction of inhalation technique was effective in pulmonary function. For instance, the study by BooskAbadi et al. showed that proper technique instruction to asthmatic patients could improve bronchodilator responses, such as an increase in PEFR.^17^Consistently, in the study AL Amoudi et al. conducted on 106 patients above 13 years old, proper instruction improved PEFR.^22^

The results of the current study revealed that learning the proper usage of MDI, with or without spacer, could increase the PEFR. A similar study showed that proper instruction both with and without spacer could increase the Forced Expiratory Volume (FEV1) among the asthmatic patients, too.^23^Since both methods have the same effects on the patients’ pulmonary function, every one of them can be recommended to the asthmatic patients considering their conditions. According to this study and other studies conducted previously, the most common problem among the asthmatic patients is coordination between inhalation and actuation. Therefore, the patients can be trained regarding utilization of spacer because it does not require coordination between inhalation and actuation. The positive effect of using spacer connected to MDI has been approved in several studies. One study showed that using MDI connected to spacer was associated with lower drug residual in oral pharyngeal space. In addition, MDI connected to spacer has been proved to be superior to nebulizer.^17^ Considering the results of the previous studies and the present one, it can be concluded that if asthmatic patients are adequately trained regarding the proper utilization of inhaler, they can choose an elective B agonist drug, such as salbutamol, to control asthma either with or without spacer.

## Conclusion

In conclusion, the most common way of controlling asthma is using MDIs, but instruction of inhaler usage techniques has not received much attention yet. Hence, the asthmatic patients’ inhalation techniques are needed to be improved. In doing so, the asthmatic patients can be trained regarding the correct application of inhalation MDIs. In this way, effective steps can be taken toward proper usage of inhalation drugs and improvement of the patients’ skills. 
